# Secondary Flexor Tendon Reconstruction: Protocol for a Systematic Review and Meta-Analysis

**DOI:** 10.29337/ijsp.176

**Published:** 2022-07-04

**Authors:** Rituja Kamble, Rushabh Shah, Ailbhe L. Kiely, Grant S. Nolan, Jason Wong

**Affiliations:** 1 University Hospitals Plymouth NHS Trust, Derriford Road, Crownhill, Plymouth, Devon, PL6 8DH, GB; 2 Manchester University NHS Foundation Trust, Cobbett House, Manchester Royal Infirmary, Oxford Rd, Manchester M13 9WL, GB; 3 Lancashire Teaching Hospitals NHS Foundation Trust, Royal Preston Hospital, Sharoe Green Ln, Fulwood, Preston PR2 9HT, GB

**Keywords:** Flexor Tendon, Secondary Reconstruction, Tendon Injuries, Tendons, Range of Motion, Articular, Tendon Graft, Hunter Rod

## Abstract

**Introduction:**

Flexor tendon injuries of the hand and wrist involve complete or partial severance of the tendon, and primary repair is standard treatment. In cases of significantly delayed presentation, rupture of the repair or segmental tendon loss may require 1- or 2-stage secondary tendon reconstruction where a tendon graft is used. There is a risk of poor functional outcome due to stiffness and reduced range of motion which may affect patient’s employment and activities of daily living. This study seeks to systematically evaluate the current evidence to determine outcomes of secondary flexor tendon reconstruction in terms of functional outcomes, complications, patient-reported outcome measures (PROMS) and costs.

**Methods:**

This is a PROSPERO registered study protocol for systematic review and meta-analysis of comparative and non-comparative studies. Outcomes of intrasynovial versus extrasynovial tendon grafting and seniority of the surgeon will be analysed in addition to comparing graft weaving at the wrist and palm for both single- and two-stage tendon reconstruction. The primary outcome is functional active range of motion. Secondary outcomes are complications, PROMs and resource use. A comprehensive literature search will be conducted from 2000 to present. All studies involving secondary flexor tendon repairs will be involved, without limitation on language, and will be screened by two independent reviewers. Tools to appraise the quality of study methodology and/or bias will be used (e.g., Cochrane Collaborative Risk of Bias tool) and if feasible, a random effects meta-analysis will be conducted.

**Ethics and dissemination:**

Ethical approval was not required for this study. The results of this systematic review and meta-analysis will be published in a peer-reviewed journal, and presented at both national and international conferences involving hand surgeons. The data collected will allow patients to be counselled more accurately by clinicians and may suggest areas where further research could be undertaken.

**Systematic review registration:**

PROSPERO CRD42021296009.

**Highlights:**

- Single- or two-stage secondary flexor tendon reconstruction is an important treatment modality in cases unsuitable for primary repair.
- This study seeks to systematically evaluate the current evidence to determine outcomes of secondary flexor tendon reconstruction in terms of functional. outcomes, complications, patient-reported outcome measures (PROMS) and costs.
- This is a PROSPERO registered study protocol for systematic review and meta-analysis of comparative and non-comparative studies.
- Outcomes of intrasynovial versus extrasynovial tendon grafting and seniority of the surgeon will be analysed in addition to comparing graft weaving at the wrist and palm for both single- and two-stage tendon reconstruction.
- This systematic review aims to evaluate and summarise the best current literature to determine outcomes of secondary flexor tendon reconstruction.

## 1. Introduction

Flexor tendon injuries occur when either the flexor digitorum profundus (FDP), flexor digitorum superficialis (FDS), or flexor pollicis longus (FPL) tendons are damaged in the hand or wrist. Injury to flexor tendons causes the inability to bend digits at the distal interphalangeal joint (DIPJ) or proximal interphalangeal joint (PIPJ). This commonly occurs following a sharp injury. The incidence of flexor tendon injuries in the United Kingdom is estimated at 4.8/100 000 person years [1]. Injuries are more common in males and inversely related to age [2]. There is a risk of poor functional outcome following this injury, with the potential to cause detriment to the patient’s activities of daily living (ADLs), quality of life (QoL) and overall ability to resume their societal role and earn a livelihood.

Management of a flexor tendon injury is surgical repair performed within 4 days following the injury [3]. In the event of a significantly delayed presentation (where primary repair is not possible), rupture of the repair with scarred ends of more than 1 centimetre in length, or segmental tendon loss, secondary flexor tendon reconstruction is considered [4]. This may be performed as a single- or two-stage procedure, the choice of which is dependent on the integrity of the pulley system and overlying soft tissue coverage. A single-stage approach is utilised if the finger or hand has adequate passive motion of all joints, a well healed wound without excessive scarring and an intact pulley system, along with a neurovascularly intact digit. In this type of reconstruction, the injured portion of the flexor tendon is removed, and immediately replaced with a free tendon graft. Donor tendons should have a similar diameter to the recipient tendon, and common donor tendons include palmaris longus, plantaris, extensor digitorum longus of the toes, a slip of FDS and extensor indicis proprius [5].

When there is an inordinate amount of scarring in the surgical bed or an inadequate pulley system, a two-stage reconstruction must be adopted. In the first stage of this technique, a silastic rod is placed in the anatomical location of the flexor tendon to recreate the flexor sheath. After the placement of this rod, the patient should undergo aggressive weekly physiotherapy for a period between 3-6 months to improve the range of motion in their joints [6]. The second stage is completed by the replacement of the silastic rod with a flexor tendon graft using donor tendons like that utilised in a single stage procedure [7].

At present, it remains to be determined whether the type of tendon graft affects postoperative outcomes following tendon reconstruction. Extrasynovial tendon grafts (located outside a tendon synovial sheath), such as palmaris longus and plantaris, are the most popular sources for donor tendons as they are easy to harvest with limited donor site morbidity. However, intrasynovial tendon grafts (located within a tendon synovial sheath), such as flexor digitorum superficialis or flexor digitorum longus, are theoretically more favourable as they have been shown to have superior biologic and biomechanical properties compared to extrasynovial tendon grafts in the synovial space [8,9,10,11]. To our knowledge, there is no literature which systematically evaluates outcomes following secondary tendon reconstruction, hence necessitating the need for the current review.

## 2. Objectives

The aim of this study is to systematically evaluate current literature to determine the outcomes following secondary flexor tendon reconstruction in adults with regards to functional outcomes, complications, resource use and patient-reported outcome measures (PROMs).

### 2.1 Primary objectives

The primary objective is to analyse outcomes of repairs using intrasynovial versus extrasynovial tendon grafts for both single and two-stage tendon reconstructions.

### 2.2 Secondary objectives

Secondary objects are to analyse outcomes relating to seniority of the surgeon and comparing graft weaving at the palm and wrist. The data collected will allow patients to be counselled more accurately by clinicians and may suggest areas where further research could be undertaken.

## 3. Methods

### 3.1 Protocol registration

This protocol has been registered in the Prospective Register of Ongoing Systematic Reviews (PROSPERO) with registration number CRD42021296009 and has been reported in accordance with the Preferred Reporting Items for Systematic Reviews and Meta-Analysis Protocols (PRISMA-P 2015) [12] (Additional file 1). The methodology of this review will be according to the Cochrane Handbook for Systematic Review of Interventions [13]. The final review will be reported following the PRISMA statement and the Meta-Analysis of Observational Studies in Epidemiology (MOOSE) guidelines [14].

### 3.2 Eligibility criteria

Selection of studies will be in accordance with the following criteria: participants, intervention, outcome measures, and study design. No limitations will be placed on peer review status or language of publication.

#### 3.2.1 Participants and population

We will include clinical data from adult patients (>16 years old) with conditions requiring secondary flexor tendon reconstruction. Studies will be included if >90% of the participants fulfil the inclusion criterion or if results for eligible participants are reported separately as subgroups. Patients who have suffered amputations or replants, undergone acute or delayed primary flexor tendon repair, or flexor tendon tenolysis, will be excluded. Additionally, data from animal studies will be excluded.

#### 3.2.2 Intervention

We will report studies that report outcomes in patients undergoing secondary flexor tendon reconstruction. Secondary flexor tendon reconstruction is performed in cases of failed primary tendon repair; where the patient presents at least 3 to 4 weeks after the initial injury or in cases with extensive tissue loss where primary tendon repair would not be appropriate. Secondary flexor tendon repair may be conducted in 1 or 2 stages depending on the extent of the tendon injury, the integrity of the pulley system and the degree of soft tissue coverage. A single-stage approach is utilised if the finger or hand has adequate passive motion of all joints, a well healed wound without excessive scarring, and a neurovascularly intact digit. However, when there is an inordinate amount of scarring in the surgical bed or an inadequate pulley system, a two-stage approach must be adopted. Patients who have undergone flexor tendon tenolysis, tendon transfer or prosthesis will be excluded.

#### 3.2.3 Comparators

The main objectives will be to compare outcomes of secondary repair techniques. The primary objective will be analysing outcomes of intrasynovial versus extrasynovial tendon grafting. Secondary objectives will be analysing outcomes relating to seniority of the surgeon and repairs involving graft weaving at the wrist versus the palm.

#### 3.2.4 Outcomes

The primary outcome will be functional active range of motion, expressed categorically using a validated tool (e.g., adjusted Strickland score [16], total active motion [TAM] [17], Buck-Gramcko score [18], LaSalle score [19]). Function will also be assessed through grip strength and punch strength where provided. Results from two different scoring systems will not be combined unless scores can be recalculated from raw data requested from the study authors.

Secondary outcomes will be complications (e.g., rupture, adhesions requiring tenolysis, infection, wound complications, complex regional pain syndrome [CRPS]), operative time (time between patient arriving and leaving theatre), resource use, and patient reported outcome measures (e.g., Patient Evaluation Measure [PEM] [20], Michigan Hand Questionnaire [MHQ] [21], Disabilities of the Arm, Shoulder and Hand [DASH] [22] or similar).

Primary and Secondary outcomes will be measured at short (<3 months), medium (3–12 months), and long-term (>12 months) follow up periods.

#### 3.2.5 Study design

Eligible studies will be both interventional and observational in nature. All comparative and non-comparative studies which report outcomes from patients undergoing secondary flexor tendon reconstruction will be included. We will exclude letters, reviews, case reports and case series with fewer than 3 patients.

#### 3.2.6 Setting

Studies performed in specialist Plastic Surgery, Orthopaedic or dedicated hand centres will be included. Patients treated outside of these settings by non-specialists will be excluded.

### 3.3 Information sources and search strategy

The primary source of literature will be a comprehensive search of the following major electronic databases from January 2000 to September 2021:

- MEDLINE (OVID SP)
- EMBASE (OVID SP)
- CINAHL (EBSCO)
- Cochrane Library
- Clinical trial registers (e.g., ClinicalTrials.gov)
- Open Grey, Dissertation databases

A hand-search will be performed of the reference lists of included studies, relevant review articles, national practice guidelines and other relevant documents to identify cited articles not found by electronic searches. Content experts and authors who are prolific in the field will be contacted. The literature searches will be designed and conducted by the review team which includes two experienced health information specialists. The search will be performed in English, relevant articles published in other languages will be translated, and will include a combination of free text and Medical Subject Heading (MeSH) terms related to secondary flexor tendon reconstruction. No restrictions will be placed on peer-review status. An example search strategy for EMBASE is included in Additional file 2.

### 3.4 Identification and selection of studies

Following database searching, relevant articles will be exported to Mendeley library, where duplicates will be removed. Study selection will be conducted in a two-stage process. Two independent reviewers will screen the titles and abstracts of the studies against a pre-specified eligibility criterion. The screening process will be performed using Covidence [15], a web-based application for systematic reviews. Full-text articles of the included studies will be screened by the same two independent reviewers. Discrepancies between reviewers at either stage will be resolved through discussion or referral to a third reviewer if required. The search results, including abstracts, full-text articles, and record of the reviewer’s decisions, including reasons for exclusion, will be recorded in Covidence^iv^ and Microsoft Excel (Microsoft Corporation, 2018).

### 3.5 Data extraction

After all full-text articles have been selected, the two reviewers will independently extract data using a standardised data extraction form in Microsoft Excel (Microsoft Corporation, 2018), with any discrepancies resolved through discussion with a third reviewer. The data collection process will be in accordance with the Cochrane Handbook of Systematic Reviews of intervention [23]. The specific details of data to be extracted from the included studies is provided in Table 1.

**Table 1:** Details of the data to be extracted from included studies.


Study characteristics	Authors, year of publication, journal, country, study design, language Inclusion criteria, exclusion criteria

Time period of data collection	Time period in months

Patient demographics	Total number of patients Number excluded Number of males/females Mean/median age + standard deviation/interquartile range

Type of intervention	Type of graft used (extrasynovial vs intrasynovial) Type of tubing used (e.g., Silicone rod, PVC catheter) Repair technique (e.g., Hunter, Paneva-Holovich) Seniority of surgeon Graft inset (palm vs wrist) Average time between stages (weeks) Pulley reconstruction Type of rehabilitation protocol

Primary outcomes (function)	Functional active range of movement (e.g., Strickland, Buck-Gramcko, Total Active Motion (TAM) scores) Grip strength Pinch strength

Secondary outcomes	Patient reported outcome measures (DASH or similar) Complications (e.g., infection, tendon rupture, adhesions requiring tenolysis, further operations) Cost (operative time)


Where relevant, we will contact the study authors via email if data relevant to the systematic review are missing in the study report. As we do not expect authors of studies published more than 11 years ago to respond to inquiries, we will only contact authors of studies published from 2010 onwards, and only when contact details (email address) are provided. If authors fail to reply after first contact or after one reminder, we will acknowledge the missing data, and proceed with the analysis.

### 3.6 Assessment of risk of bias of included studies

The risk of bias in the selected studies will be independently examined by two review authors. Any discrepancies will be resolved through discussion or referral to a third review if required. We anticipate most of the included studies to be observational rather than randomised studies, of which some will be uncontrolled (e.g., case series of two-stage flexor tendon reconstruction). A relevant tool will be utilised to assess each study design.

Randomised controlled trials will be assessed using the Cochrane Collaborative Risk of Bias tool (RoB 2) [24], which is structured into a fixed set of domains of bias, focusing on different aspects of trial design, conduct and reporting. For non-randomised comparative studies (e.g., cohort and case control studies), the Risk of Bias in Non-randomised Studies of Intervention (ROBINS-I) [25] will be utilised. Uncontrolled studies (e.g., case series) will be assessed using a tool which has been specifically for this purpose [26]. It is formed from an adaptation of previous criteria from Pierson [27], Bradford Hills [28] and Newcastle Ottawa scale [29] modifications which converge into eight items that can be categorised into four domains: selection, ascertainment, causality, and reporting.

### 3.7 Data analysis and synthesis

To address the main review question of outcomes following secondary flexor tendon repair, the data from included studies will initially be used to build evidence tables. This will include study characteristics, context, participants, outcomes, and findings.

If two or more studies are identified reporting the same outcome, we will synthesise the data to calculate improved precision estimates of both primary and secondary outcomes using a random effects meta-analysis of proportions. This will be performed by creating crude incidence estimate of each outcome (number of events/sample size) and will be presented along with 95% CI. The results of the above will be presented in forest plots.

To determine the extent of variation between selected studies, tests of heterogeneity will be performed separately for randomised and non-randomised comparative studies. Inter-study heterogeneity will be assessed visually using the forest plot. Statistical heterogeneity will be quantified statistically using three tests. The I^2^ statistic will be used and the result will be interpreted using the definitions in the Cochrane Handbook for Systematic Reviews of Interventions^xii^. Additionally, the χ^2^ and τ^2^ statistic will be used where a p-value < 0.10 will be deemed as statistically significant for heterogeneity. Any sources of heterogeneity will be explored using subgroup analysis. The overall quality evidence will be assessed using the Grading of Recommendation Assessment, Development and Evaluation (GRADE) approach [30].

### 3.8 Additional analysis

A sensitivity analysis will be performed based on the study quality to ensure the robustness of our results in any subsequent meta-analysis. Any studies which are classified as high risk of bias will be excluded including those studies where values were imputed. If contributing studies have sufficient data, individual subgroup analysis will be performed on outcomes in the single stage group and the two-stage group. For the single stage group, this will be based specifically on the type of tendon graft (e.g., intra-synovial vs extra-synovial). For the two-stage group, this will be based on; type of tendon graft (e.g., intra-synovial vs extra-synovial); technique of repair (e.g., Hunter vs Paneva-Holovich); and type of tubing (e.g., silicone rod vs foley catheter).

### 3.9 Meta-biases

Meta-biases will be identified through looking for small study effects (or publication bias across studies). This will be identified by creating a funnel plot for each meta-analysis containing 10 or more studies. Publication bias will be assessed by inspecting a funnel plot for asymmetry and with Egger’s test [31] where appropriate, with the results considered to indicate potential small study effects when p values are < 0.10, if more than 10 studies are included.

## 4. Ethics and dissemination

Ethical approval was not required for this review as secondary data will be collected. The results of the review will be disseminated via a peer-reviewed journal, and presented at national and international conferences involving hand surgeons.

## 5. Limitations

We note there may be limitations to our study. Secondary flexor tendon reconstruction is a multi-faceted approach which is not routinely performed unless in specialist plastic or orthopaedic centres. As such there may be paucity in high quality evidence regarding this topic. Furthermore, there are surgical and patient factors affecting outcomes which are not regularly revealed in studies (for example, experience of operating a surgeon, superimposed infection, pre-existing comorbidities). In non-randomised studies these may be confounding factors. We also cannot exclude reporting bias in our collection of evidence. This may be due to selective reporting of outcomes or non-publication of unfavourable evidence. We will minimise this effect by identifying publication bias using a funnel plot and contacting authors for extra information.

## 6. Conclusion

There have been many advances in reconstructive hand surgery, however flexor tendon injuries necessitating secondary repair continue to pose a challenge to all parties involved. Moreover, the socio-economic morbidity associated with these injuries has the potential to be significant. This systematic review aims to evaluate the current evidence and establish optimal management of flexor tendon injuries with regards to secondary flexor tendon repair. This will involve determining the best operative and rehabilitative techniques for optimal functional outcome, complication rate, resource use and PROMs.

## Additional Files

The additional files for this article can be found as follows:

10.29337/ijsp.176.s1 Additional File 1. PRISMA Checklist.

10.29337/ijsp.176.s2 Additional File 2. Search Strategy for EMBASE.

## Abbreviations

***CINAHL:*** The Cumulative Index to Nursing and Allied Health Literature

***CRPS:*** Complex Regional Pain Syndrome

***DASH:*** Disabilities of the Arm, Shoulder and Hand

***EMBASE:*** Excerpta Medica Database

***FDP:*** Flexor digitorum profundus tendon

***FDS:*** Flexor digitorum superficialis tendon

***FPL:*** Flexor policies longus tendon

***GRADE:*** Grade of Recommendations, Assessment, Development and Evaluation

***MEDLINE:*** Medical Literature Analysis and Retrieval System Online

***MeSH:*** Medical Subject Headings

***MHQ:*** Michigan Hand Questionnaire

***MOOSE:*** Meta-Analysis of Observational Studies in Epidemiology

***PEM:*** Patient Evaluation Measure

***PRISMA-P:*** Preferred Reporting Items for Systematic Review and Meta-Analysis Protocols

***PROMs:*** Patient Reported Outcome Measures

***PROSPERO:*** Prospective Register of Ongoing Systematic Reviews

***PVC:*** Polyvinyl chloride

***RCTs:*** Randomised controlled trials

***RoB 2:*** Cochrane Risk of Bias 2 tool

***ROBINS-I:*** Risk Of Bias in Non-randomised Studies-of Interventions

***TAM:*** Total Active Motion

## Author Information

Rituja Kamble and Rushabh K Shah are joint first authors.
