# Supplementary material for: Secondary Flexor Tendon Reconstruction: Protocol for a Systematic Review and Meta-Analysis
Source: Int J Surg Protoc. 2022 Jul 4;26(1):49–56. doi: 10.29337/ijsp.176 (PMC9266938; doi:10.29337/ijsp.176)
Supplement: Additional File 2. — Search Strategy for EMBASE. [file ijsp-26-1-176-s2.pdf]

Search Strategy for EMBASE

1. (flexor tendon• ADJ2 (repair OR reconstruct')).ti,ab
2. "FLEXOR TENDON IN JURY"/su
3. (1 OR 2)
4. (secondary OR 2-stage OR two-stage).ti,ab
5. (3 AND 4)
6. 5 [DT FROM 2000] [Human age groups Adult 18 to 64 years OR Aged 65+ years]
7. (stag").ti,ab
8. (3 AND 7)
9. 8 [DT FROM 2000] [Human age groups Adult 18 to 64 years OR Aged 65+ years]
10. ((Hunter OR silicon) ADJ1 rod).ti,ab
11. (tendon ADJ graft*).ti,ab
12. (("flexor tendon .. ADJ1 pulley) ADJ1 (reconstruction OR repair)).ti,ab
13. (tenolysis).ti,ab
14. (11 OR 13)
15. (3 AND 14)
16. (10 OR 12 OR 15)
17. 16 [DT FROM 2000] [Human age groups Adult 18 to 64 years OR Aged 65+ years] [Humans]
